# Effects of experimental warming on *Betula nana* epidermal cell growth tested over its maximum climatological growth range

**DOI:** 10.1371/journal.pone.0251625

**Published:** 2021-05-19

**Authors:** Fabian E. Z. Ercan, Juha Mikola, Tarja Silfver, Kristiina Myller, Elina Vainio, Sandra Słowińska, Michał Słowiński, Mariusz Lamentowicz, Daan Blok, Friederike Wagner-Cremer

**Affiliations:** 1 Palaeoecology, Department of Physical Geography, Utrecht University, Utrecht, The Netherlands; 2 Kevo Subarctic Research Institute, Biodiversity Unit of the University of Turku, Utsjoki, Finland; 3 Faculty of Biological and Environmental Sciences, Ecosystems and Environment Research Programme, University of Helsinki, Lahti, Finland; 4 Department of Environmental and Biological Sciences, University of Eastern Finland, Kuopio, Finland; 5 Department of Geoecology and Climatology, Polish Academy of Sciences, Institute of Geography and Spatial Organization, Warsaw, Poland; 6 (NWO), Dutch Research Council, Den Haag, The Netherlands; Baylor University, UNITED STATES

## Abstract

Numerous long-term, free-air plant growth facilities currently explore vegetation responses to the ongoing climate change in northern latitudes. Open top chamber (OTC) experiments as well as the experimental set-ups with active warming focus on many facets of plant growth and performance, but information on morphological alterations of plant cells is still scarce. Here we compare the effects of *in-situ* warming on leaf epidermal cell expansion in dwarf birch, *Betula nana* in Finland, Greenland, and Poland. The localities of the three in-situ warming experiments represent contrasting regions of *B*. *nana* distribution, with the sites in Finland and Greenland representing the current main distribution in low and high Arctic, respectively, and the continental site in Poland as a *B*. *nana* relict Holocene microrefugium. We quantified the epidermal cell lateral expansion by microscopic analysis of *B*. *nana* leaf cuticles. The leaves were produced in paired experimental treatment plots with either artificial warming or ambient temperature. At all localities, the leaves were collected in two years at the end of the growing season to facilitate between-site and within-site comparison. The measured parameters included the epidermal cell area and circumference, and using these, the degree of cell wall undulation was calculated as an Undulation Index (UI). We found enhanced leaf epidermal cell expansion under experimental warming, except for the extremely low temperature Greenland site where no significant difference occurred between the treatments. These results demonstrate a strong response of leaf growth at individual cell level to growing season temperature, but also suggest that in harsh conditions other environmental factors may limit this response. Our results provide evidence of the relevance of climate warming for plant leaf maturation and underpin the importance of studies covering large geographical scales.

## Introduction

A warmer environment affects plant growth and metabolism, causes shifts in phenology, and alters survival and reproductive success [1, 2]. Such adjustments in vegetation are expected to be especially pronounced in northern high latitudes, where ongoing climate change leads to rapidly warming growth conditions [3]. In order to quantify and predict future vegetation dynamics, field experiments are performed in various sites across the globe. Such *in-situ* experiments generate realistic settings, where environmental background conditions are maintained, while relevant abiotic growth parameters such as temperature are adjusted [4, 5]. The majority of results from experimental plot-based studies indicate that Arctic plant species as well as plant communities are sensitive to warming, but response intensity and trends can be complex, sometimes contrasting or with no apparent change [4].

Of special interest are phenological observations as changes in the life cycle of plants have a profound impact on biotic ecosystem properties, including e.g. total biomass production and reproduction capacity [6, 7], but also on abiotic properties such as hydrology and surface albedo [8]. As phenology directly influences plant performance and fitness, it can be used to model variations in plant success and ecological potential as a result of climate change [6, 9–11]. Leaf level responses in experimental set-ups are largely quantified in traits that concern whole leaves such as timing of phenological events, total and specific leaf area and leaf chemistry, including leaf N content [6]. The ontogenetic succession of leaf growth and maturation, however, is understudied although the degree of leaf maturity is an important indication for response potential to such minor changes in growth conditions that might not be captured by using other traits [12].

Final leaf size develops during the maturation phase, i.e. once the initial cell division is completed, through lateral expansion of leaf epidermal cells [13, 14]. Analysis of epidermal cell properties is commonly done by microscopic analysis of the cuticle, which enables a detailed determination of cell size and shape [12, 15]. A very indicative feature of lateral epidermal cell ontogeny is the size of epidermal cells and the degree of sinuosity of the epidermal cell walls, whole relation is quantified as the undulation index (UI) [12, 15–17]. This microphenological trait is closely related to the prevailing air temperature during the growing season, as has been shown for dwarf birch, *Betula nana* (L.) in long-term single-site studies [12] and for downy birch, *B*. *pubescens* (Ehrh.) in spatial analysis of leaves grown in Scandinavia during individual years [15].

These time-series and spatial studies have clearly shown that the UI trait acclimatizes to the intensity of the growing season, commonly expressed as growing degree days (GDD), a cumulative sum of daily degrees Celsius reached throughout the year [18]. After the initial leaf epidermal cell division and specialization, the lateral epidermal cell expansion first leads to an increase in cell size and then successively to a higher cell circumference to cell size ratio, quantified by using the UI [13]. The final stage of development reached thus depends on the length and warmth of the available growth period: long and warm seasons lead to large and highly undulated epidermal cells, while short and cold seasons suppress full maturation [12]. The effect of GDD on UI was originally quantified as a ‘paleothermometer’ where UI of (sub-) fossil *B*. *nana* epidermis material from sedimentary archives provided growing season temperature reconstructions for episodes of past climate change [12, 17, 19]. Apart from GDD influence on UI, differences in light availability has also been documented to affect UI [20–23]. The significance of measuring the UI trait in experimental studies in the context of GDD lies in the tie-in with paleo studies and an improved insight into the plasticity, adaptation, and future change of leaf cell maturation. These aspects are hard to detangle in space-for-time substitution studies [6, 24], where the range of acclimatization and adaptation potential are not investigated. *Betula nana* today is a key-species of the low-Arctic tundra. It is commonly present in experimental sites and is one of the species that is predicted to undergo an increase or expansion in the Arctic greening and shrubification scenarios [25, 26].

In the present study, we make use of the full availability of *B*. *nana* in warming experiments to investigate the ontogenetic sensitivity of leaf growth to simulated warming in contrasting geographical regions. We apply the UI to *B*. *nana* leaves collected at Blæsedalen on Disko Island (Qeqertarsuaq) in west Greenland, to leaves collected at Kevo in northernmost Finnish Lapland [27], and to leaves collected at the *B*. *nana* relic stand of Linje Mire in northern Poland [28, 29].

The main aim of our microphenological approach is to test and quantify *B*. *nana* leaf ontogenetical adjustments and sensitivity to future warmer climate simulated in plot-based experimental set ups.

## Materials and methods

**Fig 1:**
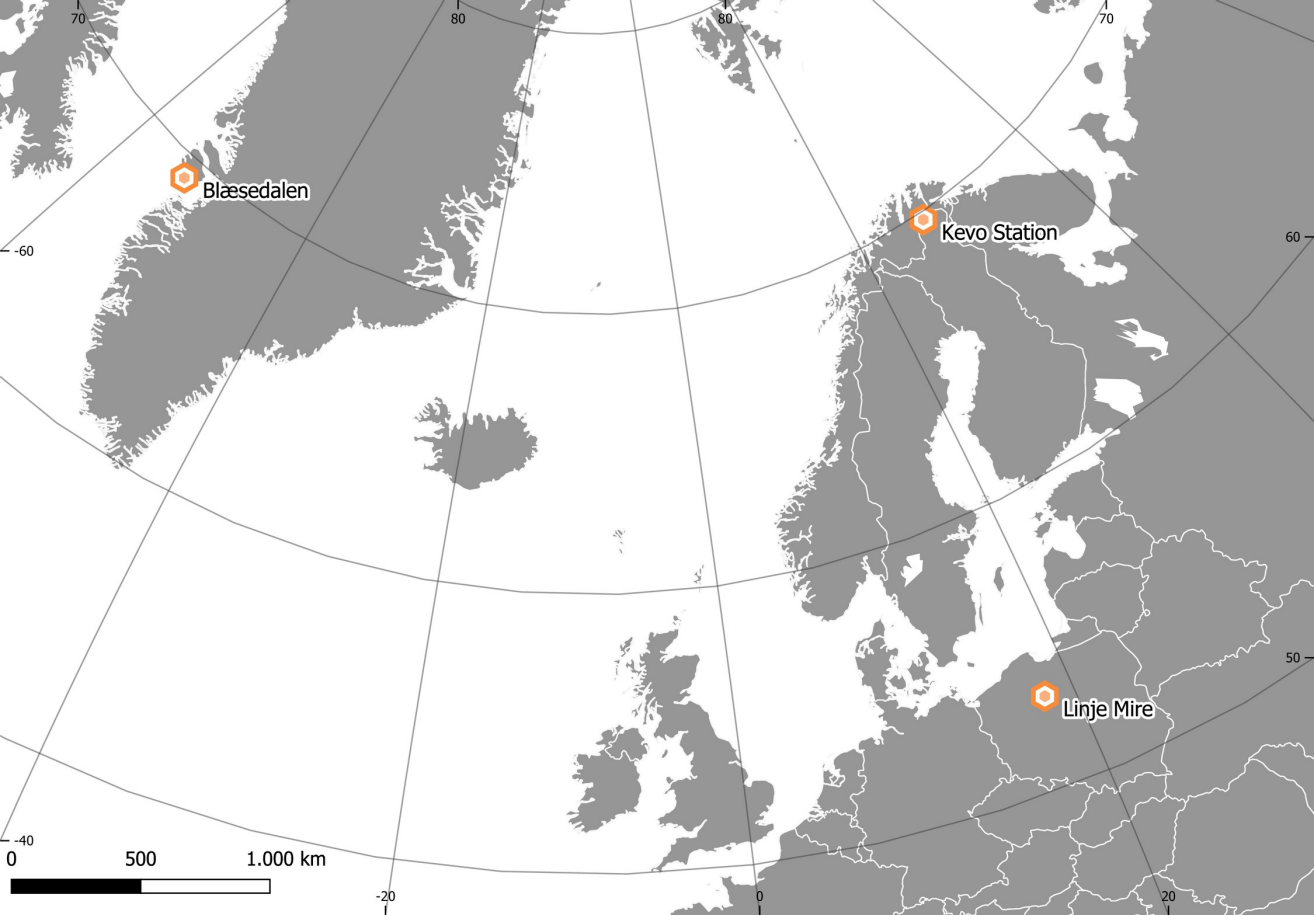
Locations of the experimental sites. Map created with QGIS (QGIS Development Team, 2021 [30]), shape files downloaded from [31].

The selected locations represent different areas of *B*. *nana* distribution, with two (sub-)Arctic sites in Finland and Greenland and a continental site in Poland (Fig 1). For all localities we studied leaf samples collected from plots with ambient temperature and those collected from plots treated with either open top chambers (OTC) or ceramic heaters to induce warming (Table 1). For each site we also studied samples from two years to facilitate a within-site comparison. By comparing years within site we are able to test the responsiveness of *B*. *nana* UI to warming under different local conditions, while the between-site comparison allows the analysis of the sensitivity of UI on a large spatial scale covering much of the distribution range of *B*. *nana*.

**Table 1:** Experimental sites and their properties.

Site	Experimental setup	Average warming above ambient (°C)	Number of used plots
Kevo station, FI	Active warming	3.3	14 (2017), 5 (2018)
Linje Mire, PL	OTC	1.5	6 (2016), 5 (2018)
Blæsedalen, GN	OTC	2.5	6 (2013), 5 (2017)

### Experimental sites

#### Greenland

**Fig 2:**
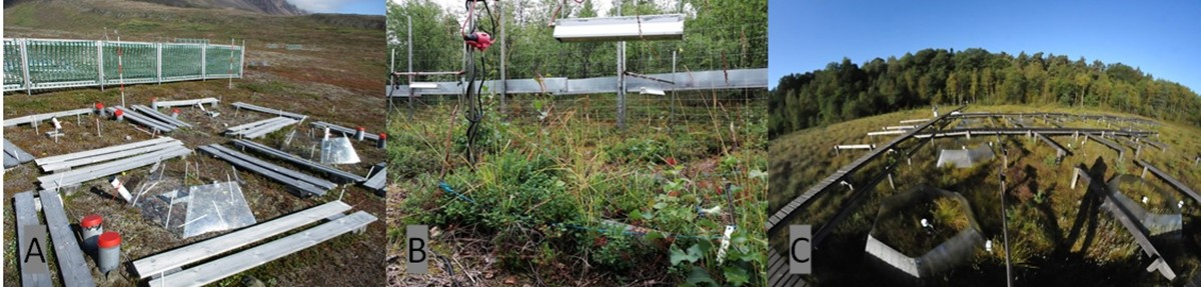
*In-situ* warming experiments: **(A)** CENPERM OTC set-up on Disko Island, Greenland **(B)** open-air warming experiment in Kevo, Finland **(C)** CLIMPEAT OTC in Linje Mire, Poland.

The CENPERM OTC set-up, see Fig 2A, is located at a mesic tundra site in the Blæsedalen valley, Qeqertarsuaq/Disko Island, West Greenland (69°16 N 53°27 W) [32]. The leaves available to this study were sampled in 2013 and 2017. Disko Island is a large island off the west coast of Greenland. It is located near the transitional zone between the low and high Arctic. The study site is a tundra/dry mixed shrub heath, dominated mainly by *B*. *nana*, *Vaccinium vitis-idaea*, *Empetrum nigrum*, *Salix glauca*, *Cassiope tetragona*, and lichens. The mean annual temperature at Blæsedalen is -3°C (1992–2012) with an average precipitation of 436mm per year (1991–2004). The OTC set-up realizes an average warming of 2.5°C and compared to ambient surface air temperatures in spring and summer [33]. The experiment was also designed to measure active layer-permafrost interactions, involving a plot treatment of shrub removal and a plot treatment facilitating extra snow cover. These treatments are not used in this study.

#### Finland

The Kevo open-air warming experiment, see Fig 2B, is situated at the Turku University Kevo Subarctic Research Institute in the northernmost Finnish Lapland (69°45.4 N, 27°00.5 E) [27]. *Betula nana* was not originally growing in the experimental site but was planted in the experimental plots as cloned plantlets of three *B*. *nana* genotypes in 2016 [27]. The leaves from these plantlets were sampled in 2017 and 2018. Apart from leaves from the warming experiment, annual monitoring of natural leaf microphenology is available in Kevo since 1996. Kevo lies within the low Arctic, or Subarctic and is characterized by a relatively mild climate. The locality is situated in the mountain birch *Betula pubescens* subsp. *czerepanovii* forest-tundra ecotone, with the local Scots pine *Pinus sylvestris* tree line 60 km to the south. The mean annual temperature at Kevo was -0.3°C in 2017 and 0.5°C in 2018, with a precipitation of 490 mm and 375 mm, respectively. The experimental set-up included ten ambient (control) plots and ten plots, where green metal plates (mimicking plant leaves) were heated to approximately 3.3°C above ambient temperature using real-time temperature measurements and microprocessor-based control of infrared ceramic heaters [27]. During 2016–2018 growing seasons, warming led to approximately 2.3°C warmer moving air and 1.2°C warmer soil in the heated plots [27]. The experiment also contained—in a fully factorial 2 × 2 design—a herbivory reduction treatment with ten plots of natural insect herbivory and ten plots with reduced insect herbivory [27]. The experiment also included a mix of plots with altered and natural herbivory regimes. In 2018, due to extreme temperatures, only the surviving plots (*n* = 5) were used.

#### Poland

The CLIMPEAT OTC set-up, see Fig 2C, is located at the nutrient poor fen Linje Mire at the border between a moraine hill and a sandur with a system of dunes, close to the northern Polish city of Bydgoszcz (53° 11 N, 18° 18 E) [34]. The leaves used in this study were sampled in 2016 and 2018. Linje Mire is particularly interesting because it is the only location in lowland Poland that maintains a glacial relict population of the arctic shrub *B*. *nana*, that has been growing in the area since the Allerød. The bog is dominated by *Sphagnum* and surrounded by a mixed forest. The mean annual temperature of the region is 8.5°C with an average precipitation of 540mm per year (1981–2010, Institute of Meteorology and Water Management-NRI). The site is located at an intersection for oceanic and continental air masses and thus has intermediate air conditions [34]. The OTC set-up realizes a maximum average warming of 1.5°C compared to ambient temperatures [35].

### Microphenology

**Fig 3:**
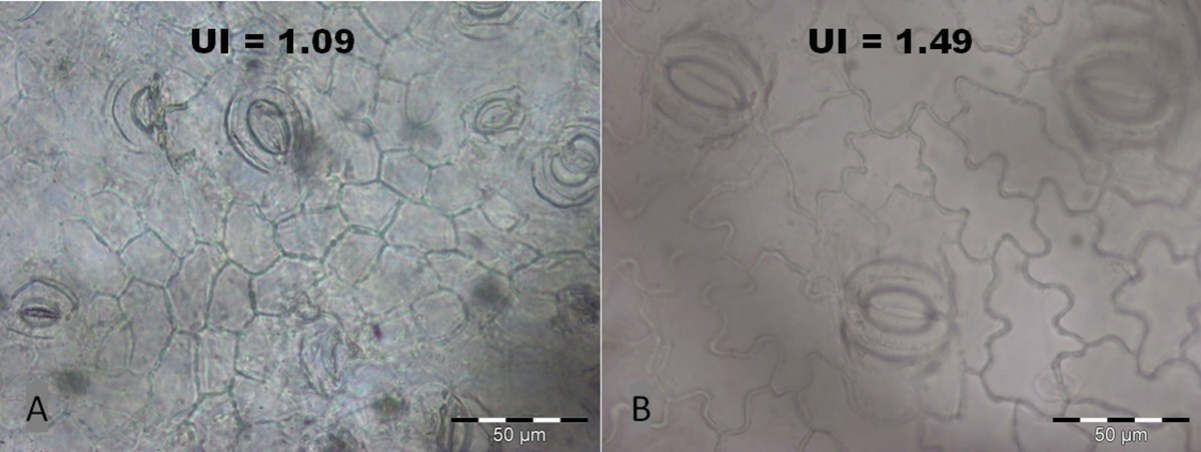
Microscopic pictures from *B*. *nana* leaf fragments. Stomata bearing alveole areas and epidermal cells with (A) low (UI: ~1.09) and (B) very high (UI: ~1.49) average cell wall undulation. Scale bar is 50 μm.

In each treatment plot, three to five leaves used in this study were sampled from one *B*. *nana* individual at the end of the growing season. From each leaf, three sections of 0.5 cm × 0.5 cm area were bleached in sodium hypochlorite (<5%) for 12–24 hours. The epidermal cell properties of three leaves per plot were then analysed using a Leica DM LB2 microscope and an AnalySIS image analysis system (Fig 3). Computer-aided analysis of epidermal and stomatal cell properties was performed using ImageJ 1.52a.

To estimate the mean epidermal cell area (CA; [μm^2^]) and epidermal cell circumference (CC; [μm]), 30 random pavement cells per leaf were analysed, avoiding cells over venation and leaf margins. From CA and CC, the undulation index (UI; dimensionless) of the epidermal cell wall was calculated following Kürschner [36].

$$UI[dimensionless]=\frac{CC}{2\pi ∙\sqrt{CA/\pi }}$$

#### Statistical comparison

The statistical significance of differences in mean UI values between the control and warming treatments were tested for each site using a paired Student’s t-test. All statistical analysis performed in R statistical software with the package ggplot2 [37, 38].

### Meteorological data

To allow comparison of cuticle analysis results to local weather conditions, data of mean daily temperatures and precipitation were collected from weather stations nearest to each sampling site.

GDD_5_ was then calculated using daily temperatures recorded throughout the growing season [27, 35, 39]. GDD covers the growing potential for vegetation in a given growing season and is expressed by the cumulative sum of degrees Celsius above a chosen base temperature [18, 40, 41]:

$${GDD}_{X}=\sum_{i=1}^{\#days}(T_{i}-X),\;T_{i}\geq X$$

where *T*_i_ is the mean temperature for day *i* in the particular site and *X* is the selected threshold temperature in degrees Celsius. For the latitudinal range covered in this study, 5 ˚C is the commonly used threshold temperature for plant growth and was thus chosen as the threshold temperature *X* [18].

## Results

**Fig 4:**
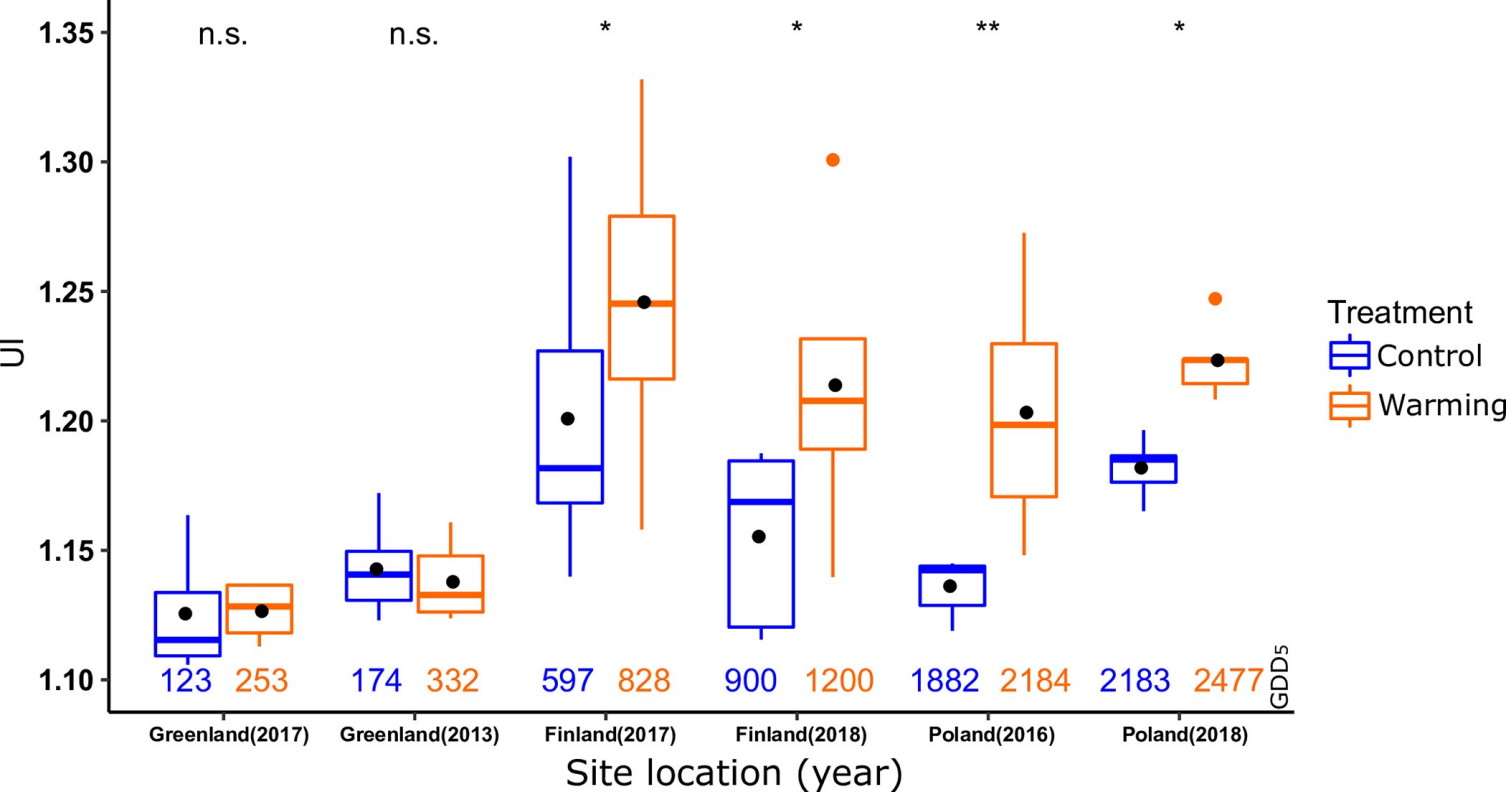
***B*. *nana* epidermal cell UI median (horizontal lines in boxplots) and mean (black dots) values for control (C) and warming (W) treatments in the different locations and years.** T-test p values are indicated with * for <0.05, ** for <0.01 and n.s. for not significant, indicating the statistical significance for differences between UI means (dots) in control and warming treatments. Numbers underneath the boxplots indicate GDD_5_ totals under which the plants have grown, green for ambient control values and red for experimental warming values.

The *B*. *nana* epidermal cell UI was compared between the ambient, or control (C) temperature and warming (W) for each site (Fig 4). The over-all UI data ranged from 1.11 to 1.33, and in Finland and Poland, warming yielded higher UI values than the ambient temperature, whereas in Greenland, no significant differences were detected.

The ambient temperature and warming treatment growing season GDD_5_ values for each experiment are shown in Table 2. The warming treatments caused a temperature increase of 1.5–3.3˚C, which lead to a GDD_5_ increase of 231–302.

**Table 2:** GDD_5_ at the moment of leaf sampling and mean UI.

Location	Ambient GDD_5_	Warmed GDD_5_	Mean UI ambient	Mean UI warming	P-value
**Finland (2017)**	597	828	1.20	1.25	0.01, *n =* 14
**Finland (2018)**	900	1200	1.16	1.21	0.03, *n =* 5
**Greenland (2013)**	174	332	1.14	1.14	0.65, *n =* 6
**Greenland (2017)**	123	253	1.13	1.13	0.93, *n =* 5
**Poland (2016)**	1882	2184 (calculated max)	1.14	1.20	0.01, *n =* 6
**Poland (2018)**	2183	2477 (calculated max)	1.18	1.22	0.02, *n =* 5

## Discussion

In this study, we compared the microphenological response of *B*. *nana* to artificial warming between experimental set-ups across the *B*. *nana* distribution. Two locations, in Finland and Greenland, are within the current distribution in the low-Arctic, while the third location in Poland is an isolated glacial relict stand [34, 42].

The ratio between cell circumference and cell area is summarized as the UI of the epidermal cells. The experiments in Finland and Poland consistently show the expected response of higher UI in the warming treatment compared to the control treatment in both individual years studied (Fig 4). This temperature response is in line with results from previous time-series data for *B*. *nana* collected in Kevo, Finland [12, 17] and studies of *B*. *pubescens* and mountain birch hybrids over a latitudinal temperature gradient in Scandinavia that both document the strong positive correlation between GDD and UI [15]. Further experimental evidence of the generality of the observed temperature response comes from climate chamber trials, where *B*. *pendula* UI values increased significantly with increasing chamber temperatures of 12°C, 20°C and 30°C in multiple weeks treatments [43].

The diverging trends observed in the experiments performed in Greenland provide interesting information on the limits in leaf growth under extremely low temperature conditions. Compared to Finland and Poland, in Greenland the GDD_5_ values were very low, ranging between ambient 123 and 332 in the warming treatment. This means that the cumulative growing season temperature available to *B*. *nana* growth is only a fraction of what is available in the other sites. In both years on Greenland, 2013 and 2017, the difference between treatments was not significant. It has earlier been shown that the growth response of plants to warming in experiments can be reduced or absent, probably due to other limiting factors, when performed in an extreme part of a plants habitat range [44], compared to a more forgiving area. In the study by Hobbie et al. [44], no vegetation and shrub biomass responses to environmental change were found in a more extreme site in Zackenberg, Greenland, while there was a biomass response in a less extreme site in Toolik, Alaska.

That temperature alone, however, does not fully govern UI development is also revealed by a comparison of data from Finland and Poland in our study. The experiment at Linje, Poland yielded lower UI values than the experiment in Kevo, Finland, although the yearly GDD is remarkably higher in Poland. This discrepancy has to be attributed to environmental conditions other than temperature, like nutrient deficiency in the Linje mire fen that create sub-optimal conditions for UI development [34, 45]. The influence of light induced UI changes, as described by Thomas *et al*. (2003) in tobacco and used in phytolith based proxies by Dunn *et al*. (2015), can be neglected since all of the experiment designs allow for optimal *B*. *nana* light conditions [20, 23].Growth chamber experiments performed with *B*. *pendula* subjected to different nitrogen supply levels resulted in significantly reduced UI in the N-limited experiments under ambient CO_2_ [43] accompanied by reduced total shoot dry weight (DW g g^-1^) measured on the same plants [46]. These studies also suggest nutrient availability as an additional stress factor for overall leaf expansion and maturation [43]. In Kevo, the UI values were lower in 2018 than in 2017 although summer 2018 had higher GDD than summer 2017. Kevo experienced extreme drought in July 2018 [27], which either suppressed leaf epidermal cell maturation [12, 47] or induced an early leaf shed during the maximum drought phase followed by a second leaf generation after precipitation was received in the later part of the growing season. In the latter case, the studied samples will have had a shorter growth period which did not allow for maximum leaf expansion by the end of the 2018 growth season. That drought has an negative effect on epidermal cell expansion, however, has been shown in field studies with laurel oak (*Quercus laurifolia*) where epidermal cell expansion is strongly supressed by low precipitation amounts received during the growing period [48]. Which of these potential causes however ultimately led to the diverging results observed between the individual years in Kevo, is difficult to disentangle and required more studies on the role of drought stress in arctic vegetation.

Previous studies have highlighted the need for more inclusive, unified and geographically widespread monitoring efforts to better resolve the interacting effects of warming and other local and regional ecological factors [4, 6]. In our study, we covered a large geographical area by carrying out the same analysis with plant material collected from comparable experiments in different locations. This approach revealed the potential restrictions imposed by local habitat and temperature ranges for plant physiological responses to warming.

The Polish and Finnish experiments showed that the epidermal UI in *B*. *nana* increases under elevated temperatures from temperate to low Arctic regions. Such plasticity and sensitivity to a subtle, but relevant, increase in temperature indicate that *B*. *nana* has the necessary physiological reactiveness to undergo enhanced plant performance under future warmer climate. However, this potential may not emerge in more extreme environmental conditions of *B*. *nana* distribution, as shown in the Greenland experiment. The response of *P*. *sylvestris* survival and growth to increasing GDD in tree line conditions was recently shown to depend heavily on soil fertility [49]. It is possible that water and nutrient availability also restricts the response of *B*. *nana* leaf cell development, and for this reason, no warming effect was found in Greenland. In sites with enhanced plant performance, higher temperatures will likely lead to plant communities with higher number and larger size of *B*. *nana* as well as expansion of *B*. *nana* into previously unsuitable areas [50–54]. This does not hold for the relic site of Linje Mire, Poland, however, as the environment involves species that would outcompete *B*. *nana* outside the refugium area.

To conclude, we found further evidence that *B*. *nana* can react to a warmer environment in terms of plant performance, which in our case was reflected in microphenology, i.e., in the UI of leaf epidermal cells. As this reaction to temperature is produced within one leaf generation, it is responsive to yearly weather variation and sensitive enough for representing growing season intensity. The potential of *B*. *nana* as one of the key plant species of tundra to increase its performance under climate warming underlines the probability of the Arctic greening scenario. Shrub expansion in the High Arctic is projected to accelerate. However, our results suggest that under the more extreme conditions of the species’ distribution range towards the High Arctic, where other limiting factors might still be at play, only rudimentary increase in *B*. *nana* growth may occur in moderate (~3°C) warming scenarios. Although, these limiting factors such as nutrient availability, soil humidity, shade, symbiotic relations, and growing season changes will change along with a warming climate in general, encouraging further combined experiments.
